# Predicting Disease-Free Survival With Multiparametric MRI-Derived Radiomic Signature in Cervical Cancer Patients Underwent CCRT

**DOI:** 10.3389/fonc.2021.812993

**Published:** 2022-01-25

**Authors:** Bing Liu, Zhen Sun, Zi-Liang Xu, Hong-Liang Zhao, Di-Di Wen, Yong-Ai Li, Fan Zhang, Bing-Xin Hou, Yi Huan, Li-Chun Wei, Min-Wen Zheng

**Affiliations:** ^1^ Department of Radiology, Xijing Hospital, Airforce Military Medical University, Xi’an, China; ^2^ Department of Orthopaedics, Xijing Hospital, Airforce Military Medical University, Xi’an, China; ^3^ Department of Radiology, Jinshan Hospital, Fudan University, Shanghai, China; ^4^ Department of Radiology, Shanxi Traditional Chinese Medical Hospital, Taiyuan, China; ^5^ Department of Radiation Oncology, Xijing Hospital, Airforce Military Medical University, Xi’an, China

**Keywords:** locally advanced cervical cancer, concurrent chemoradiotherapy, multiparametric magnetic resonance imaging, disease-free survival, radiomics

## Abstract

Prognostic biomarkers that can reliably predict the disease-free survival (DFS) of locally advanced cervical cancer (LACC) are needed for identifying those patients at high risk for progression, who may benefit from a more aggressive treatment. In the present study, we aimed to construct a multiparametric MRI-derived radiomic signature for predicting DFS of LACC patients who underwent concurrent chemoradiotherapy (CCRT).

**Methods:**

This multicenter retrospective study recruited 263 patients with International Federation of Gynecology and Obetrics (FIGO) stage IB-IVA treated with CCRT for whom pretreatment MRI scans were performed. They were randomly divided into two groups: primary cohort (n = 178) and validation cohort (n = 85). The LASSO regression and Cox proportional hazard regression were conducted to construct the radiomic signature (RS). According to the cutoff of the RS value, patients were dichotomized into low- and high-risk groups. Pearson’s correlation and Kaplan–Meier analysis were conducted to evaluate the association between the RS and DFS. The RS, the clinical model incorporating FIGO stage and lymph node metastasis by the multivariate Cox proportional hazard model, and a combined model incorporating RS and clinical model were constructed to estimate DFS individually.

**Results:**

The final radiomic signature consisted of four radiomic features: T2W__wavelet-LH_ glszm_Size Zone NonUniformity_, ADC__wavelet-HL-first order_ Median_, ADC__wavelet-HH-glrlm_Long Run Low Gray Level Emphasis_, and ADC__wavelet _LL_gldm_Large Dependence High Gray Emphasis_. Higher RS was significantly associated with worse DFS in the primary and validation cohorts (both p<0.001). The RS demonstrated better prognostic performance in predicting DFS than the clinical model in both cohorts (C-index, 0.736–0.758 for RS, and 0.603–0.649 for clinical model). However, the combined model showed no significant improvement (C-index, 0.648, 95% CI, 0.571–0.685).

**Conclusions:**

The present study indicated that the multiparametric MRI-derived radiomic signature could be used as a non-invasive prognostic tool for predicting DFS in LACC patients.

## Introduction

Cervical cancer is one of the most frequent malignancies in women, with over 604,000 new cases annually worldwide, associated with 342,000 deaths in 2020 (1). For patients diagnosed with locally advanced cervical cancer (LACC), concurrent chemoradiotherapy (CCRT) including pelvic external beam radiotherapy (EBRT), cisplatin-based chemotherapy, and brachytherapy, was the primary choice. However, about 1/3 patients suffer treatment failure; they experience unnecessary treatment-related complications and low locoreginal control rates, which worsen the prognosis (2). Usual clinical features and standard exploitation of imaging fail to deliver actionable predictive models with sufficient accuracy in cervical cancer (3, 4). Improving the patients’ risk stratification in order to individualize the treatment or surveillance schemes in cervical cancer patients would fulfill an unmet clinical need.

Magnetic resonance imaging (MRI) is recognized as the first-line image modality for diagnosing, staging, treatment planning, treatment response evaluating, and monitoring during the whole process for LACC patients (5, 6). Radiomics is an emerging field that reflects spatial and temporal heterogeneity of tumors, *via* the extraction of high-dimensional quantitative features from clinically accessibly commonly performed medical images using automated data mining algorithms, with the aim to support clinical decision-making (7–9). Previous radiomic studies in cancer have shown the potential to discover hidden information that was inaccessible with single-parameter approaches (9). For early-stage cervical cancer patients who underwent radical hysterectomy, radiomic features could predict patients’ survival with high accuracy (10). Nevertheless, whether the multiparametric MRI-derived radiomic features could be used to predict survival in LACC patients underwent CCRT remains uncertain.

Therefore, the aim of this study was to develop a radiomic signature by pretreatment MRI and evaluate the performance of different models to predict DFS in LACC patients.

## Methods

### Patients

The hospital ethics review board approved this study; written informed consent was not required for this retrospective study. All procedures performed in the study involving human participants were in accordance with the 1964 Helsinki declaration and its later amendments.

This retrospective study included patients with biopsy-confirmed locally advanced cervical cancer from three tertiary centers in different parts of China (Xijing Hospital, Shanxi Traditional Chinese Medical Hospital, and Jinshan Hospital) between October 1, 2014, and December 1, 2017. The patients’ baseline demographics, laboratory test results, pretreatment MRI images, pathological results, and survival outcome were reviewed. All patients were enrolled with strict inclusion and exclusion criteria, which are shown as follows:

Inclusion criteria

1) primary cervical cancer confirmed *via* biopsy;

2) locally advanced disease (Federation of Gynecology and Obstetrics [FIGO] stage IB-IVA) determined based on pretreatment MRI of the pelvis;

3) patients underwent pelvic MRI scans within a 2-week period before CCRT started;

3) the largest diameter of the cervical mass was 1.0 cm or larger;

4) age of 18–75 years;

5) no other treatment before MRI scan;

6) finished the entire CCRT treatment.

Exclusion criteria

1) patients with a history of cancer <5 years;

2) patients with insufficient clinical and/or follow-up data.

**Figure 1:**
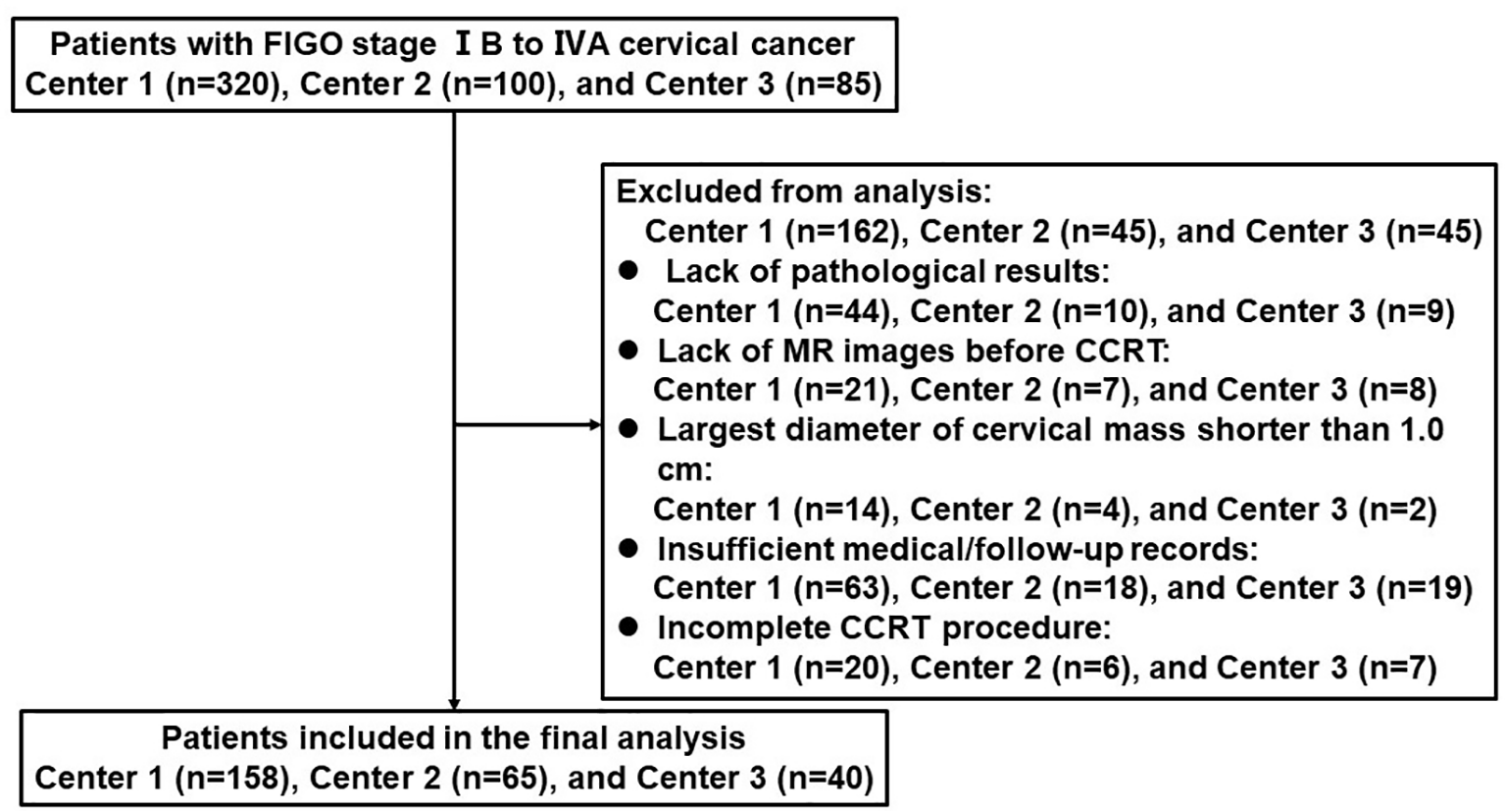
Flow diagram of patient enrollment.

Finally, 263 patients were recruited in this study. **Figure 1** displays the patient selection flowchart from the three hospitals. Eligible patients were randomly divided into a primary cohort (n = 178) and an independent validation cohort (n = 85) at a ratio of 2:1.

### CCRT Treatments and Follow-Up

All patients were treated with a combination of external beam radiotherapy (EBRT) and intracavitary brachytherapy (ICBT). EBRT was delivered to the whole pelvis with 15-MV photon beams at a daily dose of 2 Gy, 5 times per week, for a total dose of 50 Gy. EBRT was accompanied by concurrent chemotherapy: six cycles of weekly cisplatin (30 mg/mm^2^) in 30 patients and three cycles of 5-fluorouracil (1,000 mg/mm^2^) plus cisplatin (60 mg/mm^2^) at 3-week intervals in 18 patients. ICBT was delivered twice a week in 4 fractions with a fractional dose of 7 Gy at point A. the median overall treatment time was 59 days (range 45–71 days). The selection of the chemotherapeutic regime was individualized according to local tumor extent, pelvic lymph node involvement, and general patient condition (11).

Regular follow-up was conducted every 3 months during the first 2 years after treatment, 2 times annually for 3–5 years, and once a year thereafter or as clinically indicated. The endpoint of our study was DFS, which is defined as the period from the date of CCRT completion to the date of the first locoregional recurrence, distant metastasis, death, or the last visit in follow-up. Locoregional recurrences and distant metastasis were confirmed by gynecological examination and imaging modalities such as CT, MRI, and PET/CT. Available information was collected from patients’ medical records.

### MR Image Acquisitions

Pelvic MRI scans were conducted before the biopsy to avoid the impact of inflammation. All patients underwent pelvic MRI protocol that include T2WI and DWI with two b values (0 and 800 s/mm^2^). ADC maps were automatically generated and included both b values in a mono-exponential decay model. The MRI images were obtained by different MRI devices at three institutions. Detailed MRI acquisition parameters are presented in **Supplementary Methods 1**.

### Radiomic Analyses

**Figure 2:**
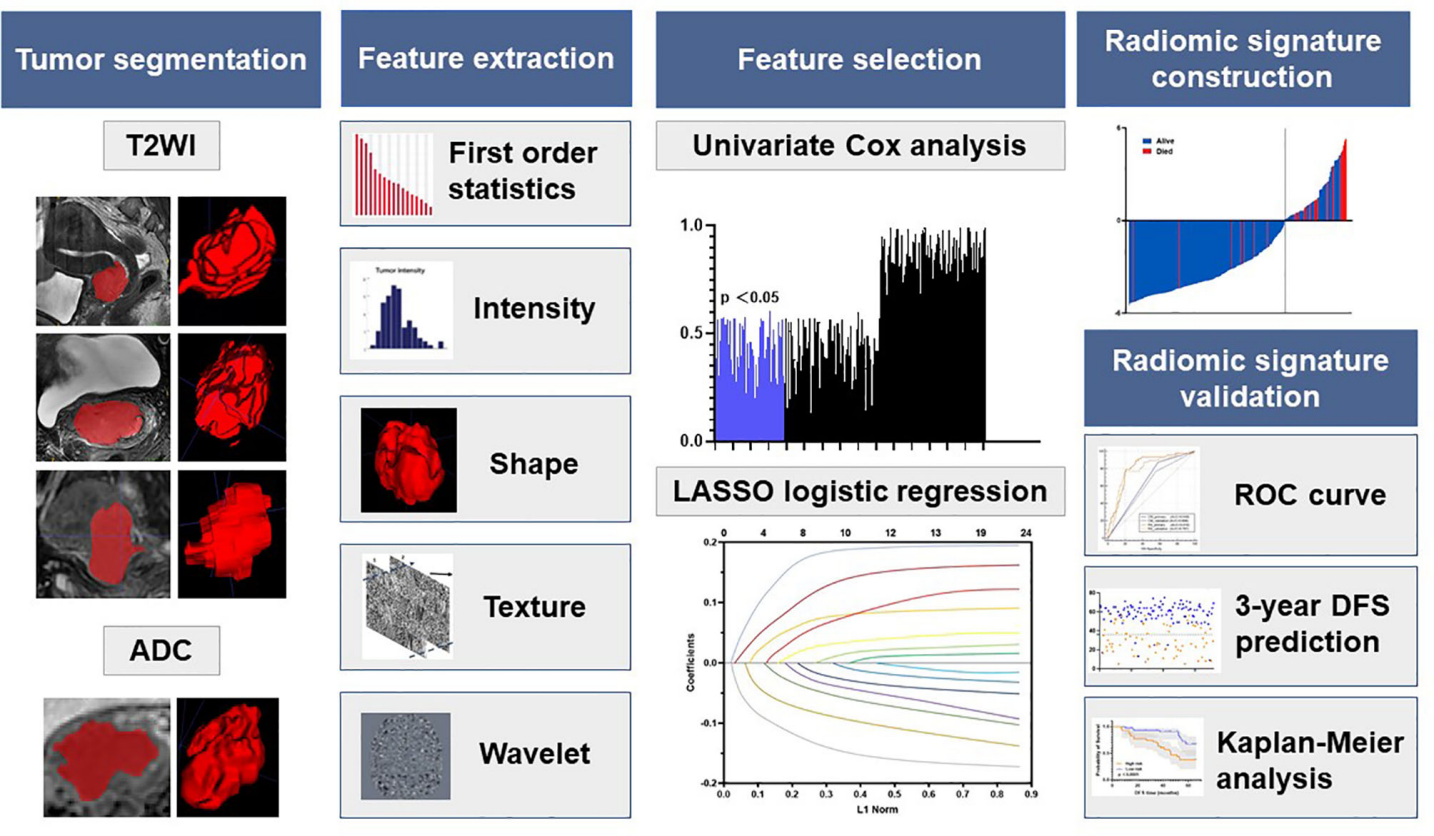
Radiomics framework of predicting the DFS of LACC patients. DFS represents disease-free survival; LACC represents locally advanced cervical cancer; ROC represents receiver operating curves.

The radiomic analysis workflow included five steps as illustrated in **Figure 2**: tumor image segmentation, radiomic feature extraction, feature selection, radiomic signature construction, and validation.

### Tumor Image Segmentation

The open-source ITK-SNAP software was used for three-dimensional manual segmentation. The regions of interest (ROIs) were delineated manually on each slice obtained in T2WI and ADC (delineated on DWI images with a b value of 800 s/mm2 and then copied to the corresponding ADC maps). Three radiologists with at least 3 years’ experience in gynecological MR imaging interpretation were chiefly responsible for the evaluation of tumor masking. To ensure reproducibility, each radiologist repeated the tumor segmentation and generation of radiomic features twice with an interval of at least 1 month, following the same procedure. To ensure the accuracy of tumor segmentation, the tumor masks were validated by a senior radiologist with 10 years of experience in segmentation result validation.

### Radiomic Feature Extraction

All images of each MRI scan for each patient were normalized separately using Z-scores to obtain a standard and normal distribution of image intensity. Then, we extracted radiomic features from T2WI and ADC respectively through an open-source package PyRadiomics, to extract 120 dimensional radiomic features of the segmented lesions. We extracted the following radiomics features: 19 first-order statistics features, 16 shape-based 3D features, 10 shape-based 2D features, 24 gray-level co-occurrence features, 16 gray-level run-length features, 16 gray-level size zone features, five neighboring gray tone difference features, and 14 gray-level dependence features. Details of the feature extraction are presented on the webpage of PyRadiomics (12). These features described the tumor information from multiscale space which incorporate the very detailed and macroscopic tumor texture patterns.

### Radiomic Signature Construction and Validation

The radiomic signature was constructed with multiparametric MRI (T2WI and ADC) based on the primary cohort. The imaging features were first normalized, and then a coarse-to-fine feature selection strategy was used to reduce the risk of bias and potential overfitting. Then, we conducted a three-step feature selection method to retain only the most robust features that are significantly associated with DFS. First, univariate Cox analysis was used to detect the associations between each feature and the DFS. All features were then ranked in ascending order according to the Cox p value, and the top 20% of the features with p<0.1 was used for the next step. Second, among these features, the Pearson correlation coefficients for each feature were then calculated. Features with |r|>0.6 were selected for the next step. Finally, the LASSO algorithm with Cox analysis was used to identify the most useful prognostic features for constructing the radiomic signature.

The potential association between the radiomic signature and DFS was initially assessed in the primary cohort and then validated in the validation cohort based on Kaplan–Meier survival analysis. The median value for the radiomic signature in the primary cohort was used as the cutoff for dividing patients into groups with high- or low-risk groups. The same cutoff value was applied to the validation cohort. The receiver-operating characteristic (ROC) curves for 1-, 2-, and 3-year DFS were plotted for each cohort, and the AUCs were quantified. Kaplan–Meier survival analysis was also performed to explore whether the radiomic signature was associated with DFS within FIGO stage subgroups for each cohort.

### Development and Validation of the Clinicopathological Model and Radiomic Signature on DFS Prediction

Among the clinicopathological factors, we firstly conducted the univariate Cox proportional hazard model to select the significant prognostic factors in the primary cohort. Then, significant factors were included in the multivariate Cox model to build a clinical model for DFS prediction.

We also evaluated whether the radiomic signature showed a superior value than the clinical model for predicting DFS in cervical cancer patients. These models were tested in the primary and validation cohorts. The prognostic performance of each model in predicting 3-year DFS was evaluated based on Harrell’s concordance index (C-index) and ROC analysis.

### Statistical Analysis

All the statistical analyses in this study were performed with SPSS v.22.0 (IBM; Armonk, NY) and R software v.4.1 (R Foundation for Statistical Computing, Vienna, Austria). Descriptive statistics were summarized as mean ± SD. Comparisons between groups were made with the t test or Mann–Whitney U test, when appropriate, for quantitative variables and with the *X* ^2^ test or Fisher’s test for qualitative variables. The interobserver agreement of feature extraction was calculated by ICC from the different radiologists’ tumor segmentation of the three radiologists. The AUC, ACC, specificity, and sensitivity with the cutoff of 0.5 and the 95% CI by the DeLong (13) method were used to assess the ability of different models to predict DFS. The Kaplan–Meier survival curve method and Cox proportional hazard model were used to analyze DFS. All tests were two-sided, and results were considered significant at p<0.05.

## Results

### Patient Characteristics

A total of 263 patients were included from three centers. The patient characteristics are displayed in **Table 1**. The mean age of the patients was 53.77 ± 8.93 years. The median follow-up time was 45.0 months (interquartile range [IQR]: 32.2–56.7 months) in the primary cohort, 43.2 months (interquartile range [IQR]: 28.6–58.5 months) in the validation cohort. The comparisons between the two cohorts showed no significant difference (p = 0.099–0.984).

**Table 1:** Characteristics of the patients at baseline.

Characteristics	Primary cohort (n = 178)	Validation cohort (n = 85)	p value
Age (years, mean ± SD)	54.28 ± 9.40	53.17 ± 9.36	0.843
SCC (ng/ml)	8.98 ± 12.10	8.74 ± 11.83	0.766
FIGO stage			0.984
IB	3 (1.69%)	1 (1.18%)	
IIA	8 (4.49%)	4 (4.71%)	
IIB	112 (62.92%)	56 (65.88%)	
IIIA	8 (4.49%)	3 (3.53%)	
IIIB	18 (10.11%)	8 (9.41%)	
IIIC	9 (5.06%)	4 (4.70%)	
IVA	20 (11.24%)	9 (10.59%)	
Histology			0.449
Squamous cell carcinoma	136 (76.41%)	61 (71.76%)	
Adenocarcinoma	34 (19.10%)	17 (20.00%)	
Adenosquamous carcinoma	8 (4.49%)	7 (8.24%)	
Tumor size			0.099
≤4 cm	107 (60.11%)	60 (70.59%)	
>4 cm	71 (39.89%)	25 (29.41%)	
Differentiation			0.797
Well	85 (47.75%)	41 (48.24%)	
Moderate	40 (22.47%)	21 (24.70%)	
Poor	53 (29.78%)	23 (27.06%)	
Lymph node metastases			0.611
Positive	45 (25.28%)	24 (28.24%)	
Negative	133 (74.72%)	61 (71.76%)	
Mean DFS time (months, mean ± SD)	42.82 ± 16.40	40.76 ± 20.17	0.452

SCC, squamous cell carcinoma antigen; FIGO, Federation of Gynecology and Obstetrics; DFS, disease-free survival.

Satisfactory inter- and intraobserver reproducibility was observed for the tumor segmentation and radiomic feature extraction (ICC>0.60) (14) when we compared results for three radiologists and results from the same radiologist at baseline and at least 1 month later.

### Radiomic Signature Construction and Validation

**Figure 3:**
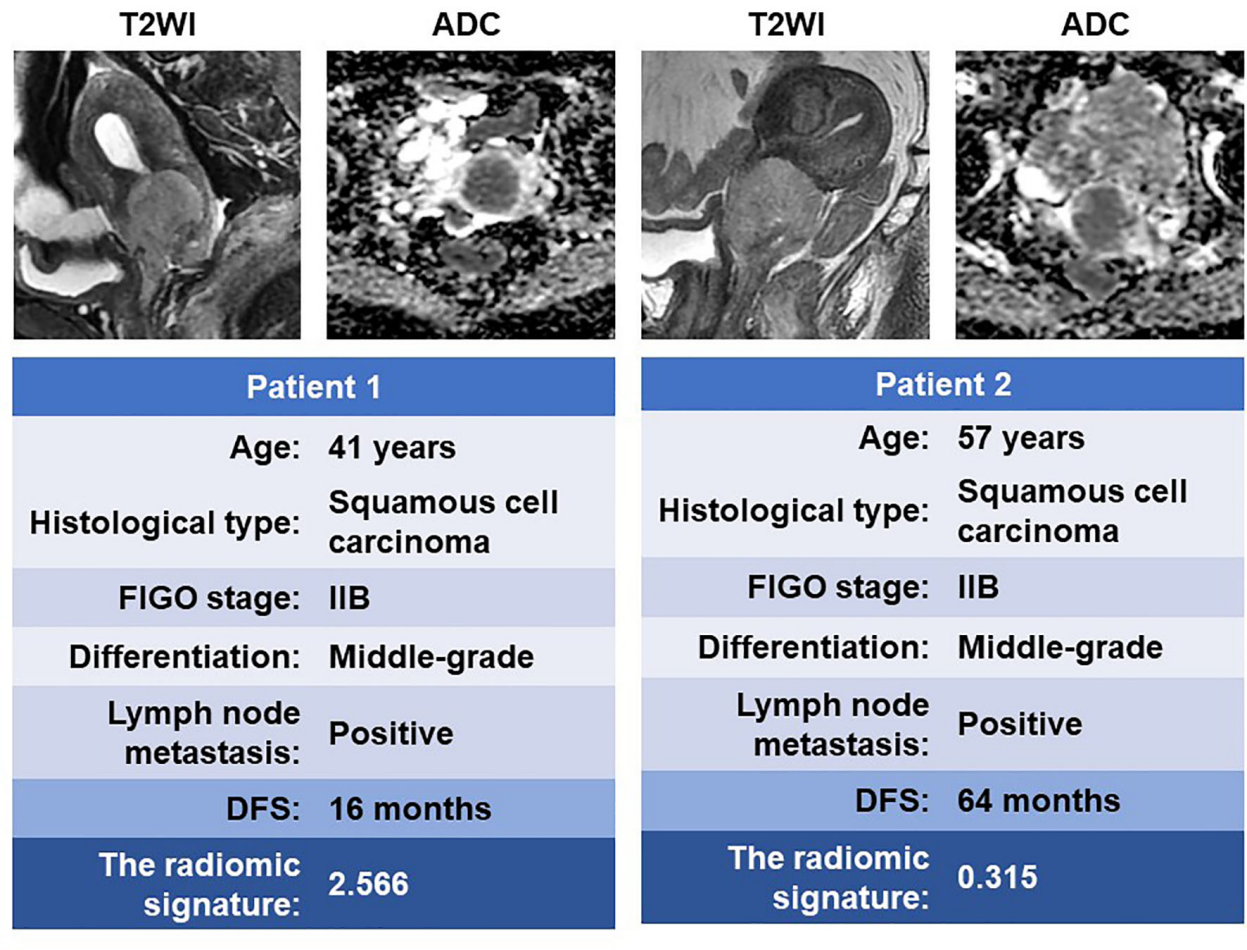
MR images of two patients with similar clinicopathological features but significantly different DFS time. DFS, disease-free survival.

A total of 4 radiomic features were selected for the RS construction (**Supplementary Table 1**). The selected features were combined into a LASSO-Cox regression model to define the radiomic signature (RS) (**Supplementary Figure 1**). For the primary cohort and the validation cohorts, patients were dichotomized into high- and low-risk groups based on the median RS of the primary cohort as the cutoff value for further analyses. **Figure 3** shows two representative patients with similar clinicopathological features, but distinctively different DFS time, due to the different risk stratification by the radiomic signature.

**Figure 4:**
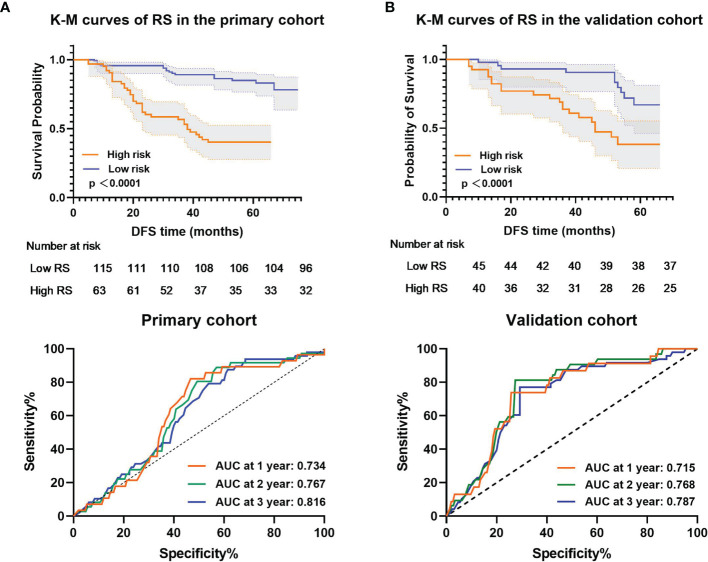
Kaplan–Meier analysis and time-dependent ROC curves of the radiomic signature. p values were calculated using a two-sided log-rank test, and AUCs at 1, 2, and 3 years were calculated to assess the prognostic accuracy within the **(A)** primary cohort (n = 178) and **(B)** validation cohort (n = 85). Shadows represent 95% CI. DFS represents disease-free survival. CI represents confidence interval.

The Kaplan–Meier survival curves confirmed a significant difference in DFS between the high- and low-RS groups (p<0.0001) (**Figure 4**, upper), with relatively high hazard ratios (HRs = 10.688) in the primary cohort (**Table 2**). In the primary cohort, the RS showed good performance on DFS prediction (C-index, 0.758; 95% CI: 0.691–0.815). In the validation cohort, the performance of the radiomic score was further confirmed (C-index, 0.736; 95% CI: 0.673–0.800) (**Table 3**). The areas under the curve (AUCs) at different follow-up times (1, 2, and 3 years) also confirmed that the RS had good prognostic accuracy in the primary and validation cohorts (**Figure 4**, lower). The hazard ratio (HR) for RS was 10.688 (p<0.001, 95% CI: 6.605–17.294) in the primary cohort and 10.880 (p<0.001, 95% CI: 6.660–17.774) in the validation cohort.

**Table 2:** Univariate and multivariable analyses between DFS, RS, and clinicopathological features in the primary cohort.

Variables	Univariate Cox regression		Multivariable Cox regression	
	HR (95% CI)	p value	HR (95% CI)	p value
Radiomic signature	10.688 (6.605–17.294)	<0.001	10.880 (6.660–17.774)	<0.001
Age	1.002 (0.974–1.032)	0.877		
FIGO stage				
I–II	–	–		
III	0.931 (0.522–1.660)	0.808	0.793 (0.432–1.455)	0.453
IVa	2.014 (1.015–3.994)	0.005	1.582 (0.566–2.971)	0.046
Histological type				
Squamous cell carcinoma	–	–		
Adenocarcinoma	3.030 (0.413–22.214)	0.276		
Adenosquamous carcinoma	1.929 (0.603–6.173)	0.269		
Differentiation				
Well	–	–		
Moderate	0.977 (0.567–1.684)	0.933		
Poor	1.520 (0.539–4.288)	0.429		
Lymph node metastases				
Negative	–	–		
Positive	2.007 (1.098–3.666)	0.002	1.599 (1.050–2.976)	0.033
SCC	1.015 (0.998–1.031)	0.088		
CA 125	1.007 (0.999–1.016)	0.088		
CEA	0.999 (0.958–1.042)	0.972		
Tumor size	1.010 (0.847–1.204)	0.914		

FIGO, Federation of Gynecology and Obstetrics; SCC, squamous cell carcinoma antigen; CEA, carcinoembryonic antigen.

**Table 3:** Model performance on predicting DFS and 3-year DFS probability.

Models	Cohorts	C-index (95% CI)	AUC (95% CI)	ACC (95% CI)	Sensitivity (95% CI)	Specificity (95% CI)
Clinical model	Primary	0.631 (0.562–0.691)	0.649 (0.574–0.719)	0.644 (0.574–0.702)	0.588 (0.437–0.713)	0.700 (0.621–0.765)
	Validation	0.603 (0.530–0.669)	0.608 (0.533–0.681)	0.610 (0.540–0.671)	0.504 (0.353–0.642)	0.715 (0.638–0.778)
Radiomic signature	Primary	0.758 (0.691–0.815)	0.816 (0.751–0.870)	0.792 (0.691–0.878)	0.792 (0.650–0.895)	0.792 (0.712–0.858)
	Validation	0.736 (0.673–0.800)	0.787 (0.726–0.845)	0.767 (0.687–0.839)	0.771 (0.627–0.880)	0.762 (0.679–0.832)
Combined model	Primary	0.648 (0.571–0.685)	0.683 (0.609–0.751)	0.682 (0.605–0.744)	0.958 (0.857–0.995)	0.407 (0.322–0.497)
	Validation	0.585 (0.511–0.637)	0.612 (0.536–0.684)	0.612 (0.533–0.679)	0.854 (0.722–0.939)	0.369 (0.286–0.458)

CI represents confidence interval. C-index represents Harrell’s concordance index, which measures the performance of the DFS prediction. AUC represents the area under the receiver operating characteristic curve, and ACC represents accuracy. AUC and ACC evaluate the performance of the 3-year DFS prediction.

**Figure 5:**
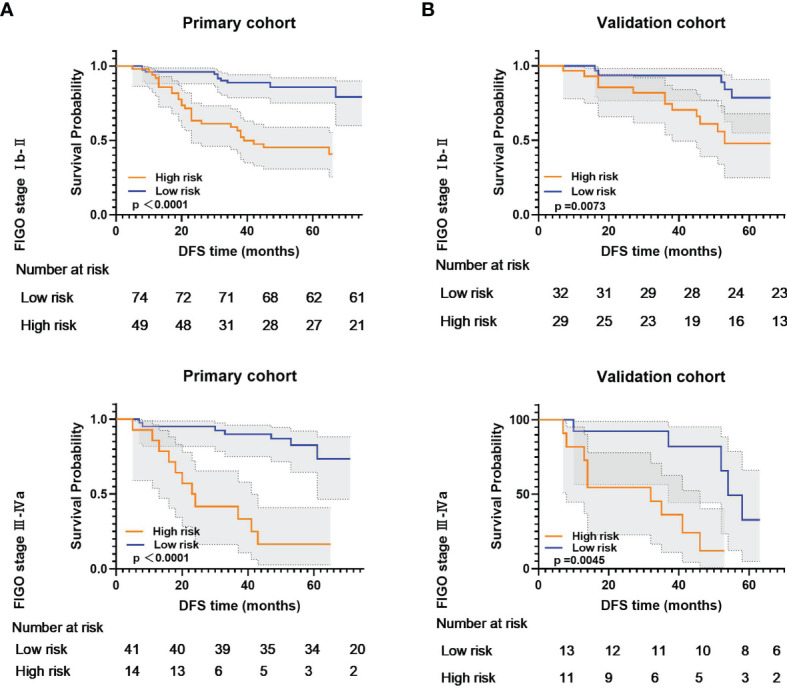
Kaplan-Meier analysis according to the radiomic signature among locally advanced cervical cancer patient subgroups in **(A)** primary cohort and **(B)** validation cohort.

Subgroup analyses further confirmed that the RS could predict prognosis according to the FIGO stage from primary and validation cohorts (**Figure 5**). These results confirmed the high prognostic accuracy of the RS.

### Performance and Validation of the Clinical Model on DFS Prediction

Only two clinical features (FIGO stage and lymph node metastasis) were selected to create a clinicopathological model (**Table 2**). This model achieved a poor performance in DFS estimation, with a C-index of 0.631 (95% CI: 0.562–0.691) in the primary cohort and 0.603 (95% CI: 0.530–0.669) in the validation cohort (**Table 3**). The combined model incorporating the RS and clinicopathological features showed no improvement in both cohorts (primary cohort, C-index: 0.648, 95% CI: 0.571–0.685; validation cohort, C-index: 0.585, 95% CI: 0.511–0.637) when compared with RS.

### 3-Year DFS Probability Prediction of Clinical Model and the RS

**Figure 6:**
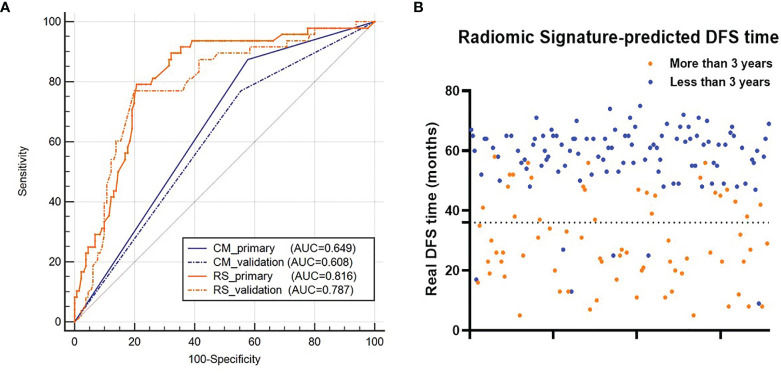
**(A)** ROC curves of the two models for 3-year DFS probability prediction. **(B)** Distribution of the DFS time for patients. The orange dots represent patients who are predicted to have DFS time longer than 3 years by the RS, and the blue dots represent patients who are predicted to have DFS time less than 3 years by the RS. RS represents radiomic signature; CM represents clinical model; ROC represents receiver-operating characteristics; DFS represents disease-free survival.

For 3-year DFS probability prediction, the clinical model achieved an AUC of 0.608 (95% CI: 0.533–0.681), sensitivity of 0.504 (95% CI: 0.353–0.642), specificity of 0.715 (95% CI: 0.638–0.778), and accuracy of 0.610 (95% CI: 0.540–0.671) in the validation cohort (**Figure 6A** and **Table 3**). The RS yielded an AUC of 0.787 (95% CI: 0.687–0.839), sensitivity of 0.771 (95% CI: 0.627–0.880), specificity of 0.762 (95% CI:0.679–0.832), and accuracy of 0.767 (95% CI: 0.687–0.839) in the validation cohort (**Figure 6A** and **Table 3**). The RS showed significant difference between patients with DFS time >3 years and <3 years (**Figure 6B**).

## Discussion

In this study, we developed and validated the prognostic value of multiparametric MR-derived radiomic features on LACC patients underwent CCRT. The results showed that LASSO-Cox-based RS had favorable predictive performance in DFS estimation than the traditional clinical model. The higher value of the RS was associated with worse outcomes, confirming that more heterogeneous tumors tended to have a poorer prognosis. Our study would help to determine whether more intensive surveillance and aggressive treatment regimens should be administered to patients with worse DFS, to assist clinical treatment and healthcare decisions.

Radiomics provided a non-invasive technique to obtain essential information of “macro-heterogeneity” underlying diagnostic, therapeutic, and prognostic information by non-invasively extracting useful imaging features from medical images (15). Radiomic biomarkers may eventually complement existing genomic and proteomic biomarkers to form a unique patient profile that informs personalized care strategies. Radiomics has been proposed for characterizing cervical cancer subtypes (16), predicting tumor staging (17), histological grading (18), and lymph node metastasis (19–21) and predicting the response to treatment either by CT, MRI alone or in combination with PET/CT (22–26).

Treatment response of cervical cancer patients varied, even patients with the same disease stage, which makes accurate prognostication essential for treatment selection (27). In the current study, we confirmed that RS was a robust predictor of DFS from a multicenter study. Furthermore, we also showed that RS was a better predictor of DFS with superior predictive potency than traditional clinicopathological features, based on larger C-index and AUCs in primary and validation cohorts. This may be because clinicopathological factors only reflect specific tumor characteristics, while radiomics based on multiparametric MRI can comprehensively and quantifiably characterize the tumor phenotype (28, 29). It is also possible that high-dimensional imaging features provide additional information, allowing radiomics to be less affected by patient distribution. Fang et al. investigated the potency of radiomic biomarkers in DFS prediction with early-stage (IB-IIA) cervical cancer patients who underwent hysterectomy by contrast-enhanced T1WI and T2WI and reported AUCs of radiomic score as 0.816 in the training cohort and 0.822 in the validation cohort (10), which was consistent and similar to our results. Sun et al. adopted T1WI- and T2WI-derived radiomic features for treatment response prediction in cervical cancer patients who underwent chemoradiotherapy; the combined model yielded an AUC of 0.998 and 0.999 in the training and validation cohorts, respectively, showing the prominent potential for treatment response evaluation and prediction of radiomic features (30). However, the AUCs in our study were 0.816 in the primary cohort and 0.787 in the training cohort, which was significantly lower than theirs. This may be caused by the different endpoints of the two studies; they defined responders as complete response or partial response within two cycles of chemotherapy regardless of the response after the final chemotherapy cycle, while we chose DFS as the endpoint of our study, which was sophisticatedly impacted by various factors. Thus, the RS constructed in our study might be better instructing personalized treatment, and the radiomic model they built was more valuable in early treatment evaluation and prediction. Although many features in MRI were significantly associated with DFS, we selected the smallest subsets of features available to achieve high accuracy for the clinical endpoint.

With excellent space definition and multiple functional imaging modalities, MRI has become the prior imaging examination in cervical cancer during the whole CCRT procedure. The clinical advantage of the extraction of MRI-derived radiomic features is that it exploits diagnostic images that are available already, so it does not require additional examinations. Moreover, extracting the RS is non-invasive and can be repeated at different time points during the whole treatment procedure. The RS provides a high-dimensional description of the intra-tumor heterogeneity. Interestingly, the ADC sequence appears to be important, as three of the four radiomic features of the RS were from ADC maps, which is consistent with previous results that ADC maps were valuable for evaluating the treatment response of various cancer types (29, 31), which also supported the indication that RS is a fairly reliable marker. Bourbonne et al. (32) and Lucia et al. (24) conducted an external validation of a multicenter study confirming the good prognostic predictive ability of ADC-derived radiomic features in prostate cancer and cervical cancer, which supported our results.

The present study has some limitations that merit consideration. Firstly, this was a retrospective study with limited sample size, and a larger prospective validation study should be conducted. The accumulation of additional patients will also allow for the collection of more information from various aspects, which can make the RS more stable and accurate. Secondly, whether imaging-derived digital biopsy features correlated with pathological biopsy results and genomic sequencing remained undefined, and molecular biology experiments should be conducted. Thirdly, although the segmentation of all images was processed by expertly trained radiologists, the use of semiautomatic segmentation tools should reduce the user dependency.

In conclusion, this present study provided a multiparametric MRI-derived radiomic signature that effectively predicted DFS in LACC patients who underwent CCRT and the RS showed superior performance than the traditional clinical model. Non-invasive MRI-derived RS showed prominent ability in risk stratification of cervical cancer patients, thus allowing radiation oncologists to select more personalized treatment regimens.

## Data Availability Statement

The raw data supporting the conclusions of this article will be made available by the authors, without undue reservation.

## Ethics Statement

The studies involving human participants were reviewed and approved by the ethics review board of Xijing Hospital. The patients/participants provided their written informed consent to participate in this study.

## Author Contributions

Conception and design: L-CW, YH, M-WZ. Collection and assembly of data: BL, ZS, FZ, Y-AL, B-XH. Data analysis and interpretation: BL, Z-LX, H-LZ, D-DW. Manuscript writing: BL, ZS, YH, M-WZ. Final approval of manuscript: all authors. All authors contributed to the article and approved the submitted version.

## Funding

We acknowledge financial support from the National Nature Science Foundation of China (Grant Numbers 81471663, 82002348) and the Key Research and Development Plan of Shaanxi Province (Grant Number: 2020ZDLSF01-01). The funders had no role in the study design, data collection, data analysis, interpretation, or writing.

## Conflict of Interest

The authors declare that the research was conducted in the absence of any commercial or financial relationships that could be construed as a potential conflict of interest.

## Publisher’s Note

All claims expressed in this article are solely those of the authors and do not necessarily represent those of their affiliated organizations, or those of the publisher, the editors and the reviewers. Any product that may be evaluated in this article, or claim that may be made by its manufacturer, is not guaranteed or endorsed by the publisher.

## Abbreviations

MRI, magnetic resonance imaging; DFS, disease-free survival; FIGO, International Federation of Gynecology and Obstetrics; LASSO, least absolute shrinkage and selection operator; PET/CT, positron emission tomography-computed tomography; T2W, T2-weighted; ADC, apparent diffusion coefficient; DWI, diffusion-weighted imaging; DICOM, Digital Imaging and Communications in Medicine; PACS, picture archiving and communication system; ROI, regions of interest; LACC, locally advanced cervical cancer; ROC, receiver-operating characteristic; AUC, area under the curve.

## References

[1] Sung H Ferlay J Siegel RL Laversanne M Soerjomataram I Jemal A. Global Cancer Statistics 2020: GLOBOCAN Estimates of Incidence and Mortality Worldwide for 36 Cancers in 185 Countries. CA: Cancer J Clin (2021) 71:209–49. doi: 10.3322/caac.21660 33538338

[2] Katsumata N Yoshikawa H Kobayashi H Saito T Kuzuya K Nakanishi T. Phase III Randomised Controlled Trial of Neoadjuvant Chemotherapy Plus Radical Surgery vs Radical Surgery Alone for Stages IB2, IIA2, and IIB Cervical Cancer: A Japan Clinical Oncology Group Trial (JCOG 0102). Brit J Cancer (2013) 108:1957–63. doi: 10.1038/bjc.2013.179 PMC3671094 23640393

[3] Rose PG Java J Whitney CW Stehman FB Lanciano R Thomas GM. Nomograms Predicting Progression-Free Survival, Overall Survival, and Pelvic Recurrence in Locally Advanced Cervical Cancer Developed From an Analysis of Identifiable Prognostic Factors in Patients From NRG Oncology/Gynecologic Oncology Group Randomized Trials of Chemoradiotherapy. J Clin Oncol: Off J Am Soc Clin Oncol (2015) 33:2136–42. doi: 10.1200/JCO.2014.57.7122 PMC4477785 25732170

[4] Kristensen GB Abeler VM Risberg B Trop C Bryne M. Tumor Size, Depth of Invasion, and Grading of the Invasive Tumor Front are the Main Prognostic Factors in Early Squamous Cell Cervical Carcinoma. Gynecol Oncol (1999) 74:245–51. doi: 10.1006/gyno.1999.5420 10419739

[5] Ward ZJ Grover S Scott AM Woo S Salama DH Jones EC. The Role and Contribution of Treatment and Imaging Modalities in Global Cervical Cancer Management: Survival Estimates From a Simulation-Based Analysis. Lancet Oncol (2020) 21:1089–98. doi: 10.1016/S1470-2045(20)30316-8 PMC7574952 32758463

[6] Westerveld H Nesvacil N Fokdal L Chargari C Schmid MP Milosevic M. Definitive Radiotherapy With Image-Guided Adaptive Brachytherapy for Primary Vaginal Cancer. Lancet Oncol (2020) 21:e157–67. doi: 10.1016/S1470-2045(19)30855-1 32135119

[7] Lambin P Rios-Velazquez E Leijenaar R Carvalho S van Stiphout RGPM Granton P. Radiomics: Extracting More Information From Medical Images Using Advanced Feature Analysis. Eur J Cancer (2012) 48(4):441–6. doi: 10.1016/j.ejca.2011.11.036 PMC4533986 22257792

[8] Aerts HJWL Velazquez ER Leijenaar RTH Parmar C Grossmann P Carvalho S. Decoding Tumour Phenotype by Noninvasive Imaging Using a Quantitative Radiomics Approach. Nat Commun (2014) 5:4006. doi: 10.1038/ncomms5006 24892406 PMC4059926

[9] Lambin P Leijenaar RTH Deist TM Peerlings J de Jong EEC van Timmeren J. Radiomics: The Bridge Between Medical Imaging and Personalized Medicine. Nat Rev Clin Oncol (2017) 14:749–62. doi: 10.1038/nrclinonc.2017.141 28975929

[10] Fang J Zhang B Wang S Jin Y Wang F Ding Y. Association of MRI-Derived Radiomic Biomarker With Disease-Free Survival in Patients With Early-Stage Cervical Cancer. Theranostics (2020) 10:2284–92. doi: 10.7150/thno.37429 PMC7019161 32089742

[11] Liu B Sun Z Ma W Ren J Zhang G Wei M. DCE-MRI Quantitative Parameters as Predictors of Treatment Response in Patients With Locally Advanced Cervical Squamous Cell Carcinoma Underwent CCRT. Front Oncol (2020) 10:585738. doi: 10.3389/fonc.2020.585738 33194734 PMC7658627

[12] van Griethuysen JJM Fedorov A Parmar C Hosny A Aucoin M Narayan NV. Computational Radiomics System to Decode the Radiographic Phenotype. Cancer Res (2017) 77:e104–7. doi: 10.1158/0008-5472.CAN-17-0339 29092951 PMC5672828

[13] DeLong ER DeLong DM Clarke-Pearson DL. Comparing the Areas Under Two or More Correlated Receiver Operating Characteristic Curves: A Nonparametric Approach. Biometrics (1988) 44:837–45. doi: 10.2307/2531595 3203132

[14] Landis JR Koch GG. The Measurement of Observer Agreement for Categorical Data. Biometrics (1977) 33:159–74. doi: 10.2307/2529310 843571

[15] Gillies RJ Kinahan PE Hricak H. Radiomics: Images are More Than Pictures, They are Data. Radiology (2016) 278:563–77. doi: 10.1148/radiol.2015151169 PMC4734157 26579733

[16] Tsujikawa T Rahman T Yamamoto M Yamada S Tsuyoshi H Kiyono Y. (18)F-FDG PET Radiomics Approaches: Comparing and Clustering Features in Cervical Cancer. Ann Nucl Med (2017) 31:678–85. doi: 10.1007/s12149-017-1199-7 28815452

[17] Mu W Chen Z Liang Y Shen W Yang F Dai R. Staging of Cervical Cancer Based on Tumor Heterogeneity Characterized by Texture Features on (18)F-FDG PET Images. Phys Med Biol (2015) 60:5123–39. doi: 10.1088/0031-9155/60/13/5123 26083460

[18] Liu Y Zhang Y Cheng R Liu S Qu F Yin X. Radiomics Analysis of Apparent Diffusion Coefficient in Cervical Cancer: A Preliminary Study on Histological Grade Evaluation. J Magn Reson Imaging: JMRI (2019) 49:280–90. doi: 10.1002/jmri.26192 29761595

[19] Song J Hu Q Ma Z Zhao M Chen T Shi H. Feasibility of T(2)WI-MRI-Based Radiomics Nomogram for Predicting Normal-Sized Pelvic Lymph Node Metastasis in Cervical Cancer Patients. Eur Radiol (2021) 31:6938–48. doi: 10.1007/s00330-021-07735-x 33585992

[20] Hou L Zhou W Ren J Du X Xin L Zhao X. Radiomics Analysis of Multiparametric MRI for the Preoperative Prediction of Lymph Node Metastasis in Cervical Cancer. Front Oncol (2020) 10:1393. doi: 10.3389/fonc.2020.01393 32974143 PMC7468409

[21] Dong T Yang C Cui B Zhang T Sun X Song K. Development and Validation of a Deep Learning Radiomics Model Predicting Lymph Node Status in Operable Cervical Cancer. Front Oncol (2020) 10:464. doi: 10.3389/fonc.2020.00464 32373511 PMC7179686

[22] Lucia F Visvikis D Desseroit M Miranda O Malhaire J Robin P. Prediction of Outcome Using Pretreatment (18)F-FDG PET/CT and MRI Radiomics in Locally Advanced Cervical Cancer Treated With Chemoradiotherapy. Eur J Nucl Med Mol I (2018) 45:768–86. doi: 10.1007/s00259-017-3898-7 29222685

[23] Bowen SR Yuh WTC Hippe DS Wu W Partridge SC Elias S. Tumor Radiomic Heterogeneity: Multiparametric Functional Imaging to Characterize Variability and Predict Response Following Cervical Cancer Radiation Therapy. J Magn Reson Imaging: JMRI (2018) 47:1388–96. doi: 10.1002/jmri.25874 PMC5899626 29044908

[24] Lucia F Visvikis D Vallières M Desseroit M Miranda O Robin P. External Validation of a Combined PET and MRI Radiomics Model for Prediction of Recurrence in Cervical Cancer Patients Treated With Chemoradiotherapy. Eur J Nucl Med Mol I (2019) 46:864–77. doi: 10.1007/s00259-018-4231-9 30535746

[25] Ferreira M Lovinfosse P Hermesse J Decuypere M Rousseau C Lucia F. [(18)F]FDG PET Radiomics to Predict Disease-Free Survival in Cervical Cancer: A Multi-Scanner/Center Study With External Validation. Eur J Nucl Med Mol I (2021) 48:3432–43. doi: 10.1007/s00259-021-05303-5 PMC8440288 33772334

[26] Li H Zhu M Jian L Bi F Zhang X Fang C. Radiomic Score as a Potential Imaging Biomarker for Predicting Survival in Patients With Cervical Cancer. Front Oncol (2021) 11:706043. doi: 10.3389/fonc.2021.706043 34485139 PMC8415417

[27] Kato S Ohno T Thephamongkhol K Chansilpa Y Cao J Xu X. Long-Term Follow-Up Results of a Multi-Institutional Phase 2 Study of Concurrent Chemoradiation Therapy for Locally Advanced Cervical Cancer in East and Southeast Asia. Int J Radiat Oncol Biol Phys (2013) 87:100–5. doi: 10.1016/j.ijrobp.2013.04.053 23920390

[28] Aerts HJWL. The Potential of Radiomic-Based Phenotyping in Precision Medicine: A Review. JAMA Oncol (2016) 2:1636–42. doi: 10.1001/jamaoncol.2016.2631 27541161

[29] Liu Z Meng X Zhang H Li Z Liu J Sun K. Predicting Distant Metastasis and Chemotherapy Benefit in Locally Advanced Rectal Cancer. Nat Commun (2020) 11:4308. doi: 10.1038/s41467-020-18162-9 32855399 PMC7452897

[30] Sun C Tian X Liu Z Li W Li P Chen J. Radiomic Analysis for Pretreatment Prediction of Response to Neoadjuvant Chemotherapy in Locally Advanced Cervical Cancer: A Multicentre Study. Ebiomedicine (2019) 46:160–9. doi: 10.1016/j.ebiom.2019.07.049 PMC6712288 31395503

[31] Liu Z Li Z Qu J Zhang R Zhou X Li L. Radiomics of Multiparametric MRI for Pretreatment Prediction of Pathologic Complete Response to Neoadjuvant Chemotherapy in Breast Cancer: A Multicenter Study. Clin Cancer Res: an Off J Am Assoc Cancer Res (2019) 25:3538–47. doi: 10.1158/1078-0432.CCR-18-3190 30842125

[32] Bourbonne V Fournier G Vallières M Lucia F Doucet L Tissot V. External Validation of an MRI-Derived Radiomics Model to Predict Biochemical Recurrence After Surgery for High-Risk Prostate Cancer. Cancers (2020) 12. doi: 10.3390/cancers12040814 PMC7226108 32231077

