# Supplementary material for: Predicting Disease-Free Survival With Multiparametric MRI-Derived Radiomic Signature in Cervical Cancer Patients Underwent CCRT
Source: Front Oncol. 2022 Jan 25;11:812993. doi: 10.3389/fonc.2021.812993 (PMC8821662; doi:10.3389/fonc.2021.812993)
Supplement: Supplementary file 1 [file DataSheet_1.docx]

**Supplementary Methods 1.** Magnetic resonance image acquisition parameters used in the present study.

The patients from Xijing Hospital were examined using the SIEMENS 3.0 T TrioTim and GE 3.0 T Discovery MR750 with the following scanning parameters: axial, sagittal and coronal T2 weighted spin-echo images (TR/TE:4000/100 ms, FOV:20*18cm, NEX:4, slice thickness:3mm, spacing:0.3 mm), and axial DWI (TR:500 ms; TE:105 ms; slice thickness: 3 mm; spacing: 0.3 mm; FOV: 230*230 mm; matrix: 128*128)

The patients from Shanxi Traditional Chinese Medical Hospital were using Phillips Ingenia 1.5 T and 3.0 T with the following scanning parameters: axial, sagittal and coronal T2-weighted spin-echo images (TR/TE:3000/100 ms, FOV:40*40cm, Matrix:200*200, Spatial resolution:1.0*1.0, Section gap:0.4mm, No. of sections:24), and axial DWI (TR/TE:3500/87 ms, FOV:30*30cm, Matrix:100*80, Spatial resolution:3.0*3.0, Slice thickness: 3mm, Section gap:1mm).

The patients from Jinshan Hospital were using GE 1.5 T OPTIMA MR 360 and GE 3.0 T Signa HDxt with the following scanning parameters: axial, sagittal and coronal T2-weighted spin-echo images (TR/TE:5000/100 ms, FOV:40*40cm, NEX:2, slice thickness:3mm, spacing:0.3 mm), and axial DWI (TR/TE:2000/87 ms, FOV:38*38cm, NEX:2, slice thickness:4mm, spacing:0.4 mm).

**Supplementary Table 1.** The name of the 4 selected radiomic features.

|  | Feature name |
| --- | --- |
| Feature 1 | T2W_wavelet-LH_glszm_SizeZoneNonUniformity |
| Feature 2 | ADC_wavelet-HL-firstorder_Median |
| Feature 3 | ADC_wavelet-HH-glrlm_LongRunLowGrayLevelEmphasis |
| Feature 4 | ADC_wavelet_LL_gldm_LargeDependenceHighGrayEmphasis |

Note: T2W represents T2 weighted; ADC represents apparent diffusion coefficient.

**Supplementary Figure 1.** Distribution of the selected radiomic features and the Radiomic Signature. The line in each figure is the linear regression of the points, and the shadow represent 95% confidence interval.
